# Constitutional Growth Delay Pattern of Growth in Velo−Cardio−Facial Syndrome: Longitudinal follow up and final height of two cases

**DOI:** 10.4008/jcrpe.v1i1.13

**Published:** 2008-08-07

**Authors:** Serap Turan, Nihal Özdemir, Tülay Güran, Figen Akalın, Teoman Akçay, Canan Ayabakan, Yüksel Yılmaz, Abdullah Bereket

**Affiliations:** 1 Marmara University, Department of Pediatric Endocrinology, Istanbul, Turkey; 2 Marmara University, Department of Cardiology, Istanbul, Turkey; 3 Marmara University, Department of Neurology, Istanbul, Turkey; +90−216 327 10 10+90−216 325 03 25serapdemircioglu@yahoo.comMarmara Üniversitesi Tıp Fakültesi Hastanesi Çocuk Sağlığı ve Hastalıkları AD Tophanelioğlu Cad. Altunizade Üsküdar−İstanbul−Turkey

**Keywords:** growth, Growth hormone deficiency, Velo−cardio−facial syndrome, 22q11.2, short stature, normal genotype

## Abstract

We report two patients with velo−cardio−facial syndrome (VCFS) who were admitted to our pediatric endocrinology clinic because of short stature and followed longitudinally until attainment of final height. Both patients followed a growth pattern consistent with constitutional delay of puberty with normal and near normal final height. Case 2 also had partial growth hormone (GH) deficiency and severe short stature (height SDS −3.4 SDS), but showed spontaneous catch−up and ended up with a final height of −2 SDS. These cases suggest that short stature in children with VCFS is due to a pattern of growth similar to that observed in constitutional delay of growth and puberty.

**Conflict of interest:**None declared.

## INTRODUCTION

Velo−cardio−facial syndrome (VCFS) is characterized by a typical facial appearance, learning disabilities, congenital heart defects, hypernasal speech, cleft palate, short stature and transient neonatal hypocalcemia. It was first described by Shprintzen et al(1) in 1978 as a distinct multiple anomaly syndrome. The specific genetic cause of VCFS is a deletion in the long arm of chromosome 22 at the q11.2 band.(2, 3) Distal deletion of 4q and partial monosomy 10p has also been reported to cause a syndrome similar to VCFS.(4, 5) However, genetic defects could not be detected by fluorescence in situ hybridization (FISH) analysis in 10% of VCFS cases.(6, 7, 8, 9, 10, 11, 12) A spectrum of phenotypes, often collectively called CATCH22 (cardiac defect, anomaly of face, cleft palate, hypoparathyroidism), is associated with heterozygous deletions of chromosome 22q11.2. This spectrum includes the overlapping entities, DiGeorge malformation complex, VCFS, conotruncal anomaly face syndrome, and isolated outflow tract defect of the heart (conotruncal heart defect: tetralogy of Fallot, truncus arteriosus, and interrupted aortic arch).

Postnatal growth deficiency and short stature are common features of VCFS reported in 36% to 67% of the patients.(13, 14, 15, 16, 17, 18, 19, 20) All patients with 22q11.2 deletion having short stature reported in a recent study were younger than 10 years of age.(21) On the other hand, only 10% of adult patients are reported to be below normal height.(13) These observations suggest that short stature in VCFS could be attributable to a growth pattern similar to constitutional delay. To our knowledge, there are no longitudinal studies describing the pattern of growth in patients with VCFS. Furthermore, partial growth hormone deficiency has been detected in 4 patients with 22q11.2 deletion.(22)

Here we report longitudinal growth data of two patients with VCFS and short stature who were followed until attainment of final height.

## CASE REPORTS

**Case 1**

This female patient was the first child of healthy, non−consanguineous parents. She was born at term after an uneventful pregnancy with a birth weight of 2800 g. At age 3 years she had an operation for club foot. At age 11 years and 4 months she was admitted to our hospital with epileptic seizure. Her weight was 28 kg and her height was 138 cm (−2 SDS and −1.9 SDS respectively by national standards). Her bone age was 9 years and 3 months (Greulich Pyle method). Midparental (target) height was 161 cm (+0.2 SDS). On physical examination she had a prominent nasal root, malar hypoplasia, long face, narrow palpebral fissures, high arched palate, long and slender fingers, scoliosis and umbilical hernia (Figure 1a). Hypernasal speech was noted. The diagnosis of hypoparathyroidism was made on follow−up, based on the low calcium (7.1 mg/dL), high phosphorus (6.2 mg/dl) and inappropriately low parathyroid hormone (32.5 pg/mL) levels. The typical facial appearance of the patient and her hypoparathyroid state led us to consider VCFS. Chromosome 22q11.2 deletion was demonstrated by FISH analysis using the specific DNA probe (Figure 2). However, it has not been possible to perform a parental FISH analysis in this particular case. Echocardiography demonstrated atrial septal defect. Conductive type hearing loss was detected on the audiogram. WISC−R score was 66 IQ. The lymphocyte subset analysis and serum immunoglobulin levels were normal. Renal ultrasound and thyroid functions were also normal. Basal ganglia calcification was not detected on cranial tomography. At follow−up, catch−up growth was noted during puberty (Figure 3). She had menarche at age of 15.5 years. The patient’s final height and weight measurements were 159.3 cm (−0.1 SDS) and 50.8 kg (−0.8 SDS) respectively at age 18 years and consistent with her target height which was 161 cm (+0.2 SDS).

**Case 2**

A 13 year−old girl was referred to our pediatric endocrinology clinic for evaluation of short stature. Parents were first cousins. She was born at term by vaginal delivery with a birth weight of 2100 g. At age 11.5 years she had a tonsillectomy/ adenoidectomy operation because of recurrent otitis media. She had learning disabilities especially in mathematics at school and was reported to have attention deficit hyperactivity disorder. She also had decreased visual sharpness. On physical examination her height was 135.8 cm (−3.4 SDS), her weight was 27.7 kg (−2.3 SDS) while her midparental (target) height was 153.2 cm (−1.2 SDS). Her bone age corresponded to 10 years (Greulich Pyle method) at the chronological age of 13 years. She had a depressed nasal bridge, hypoplastic ala nasi, long filtrum, long face, narrow palpebral fissures, hyperteleorism, high arched palate and slender fingers (Figure 1b). A high grade systolic ejection murmur was heard on the left sternal border and echocardiography revealed coarctation of aorta and double aortic arch. Small optic discs and tortuous retinal vessels were demonstrated on ophthalmologic examination. WISC−R score was 42 IQ. Diagnosis of VCFS was considered. However, no deletion in the 22q11.2 region was found by FISH and high resolution karyotype analysis was normal. The patient did not have a history of recurrent infections other than otitis media. She had normal serum calcium levels and thyroid hormone levels. A growth hormone (GH) provocative test by clonidine revealed partial GH deficiency (peak GH 6.5 ng/ml). The patient was lost to follow up for 3 years. She had undergone a cardiac operation in this time period. Her menarche had occurred at age of 14. Her serum insulin like growth factor I (IGF−I) and IGF binding protein 3 (IGFBP−3) levels were 188 and 3770 ng/mL respectively and below –2 SDS for her age. The patient’s final height at 16.5 years was 148.3 cm (−2.0 SDS), 0.8 SDS lower than her target height. However, a second growth hormone stimulation test with L−Dopa performed at this time revealed showed a normal peak serum GH level (11.7 ng/ml).

**Figure 1a:**
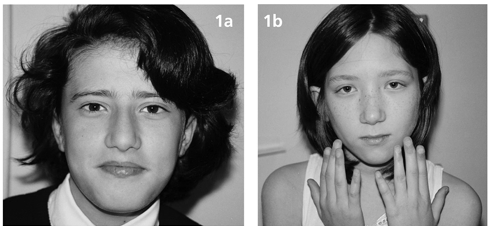
Facial appearance showing prominent nasal root, malar hypoplasia, long face, narrow palpebral fissures of case 1 (1a) and, depressed nasal bridge, hypoplastic ala nasi, long filtrum, long face, narrow palpebral fissures, hypertelorism and slender fingers of case 2 (1b).

**Figure 2:**
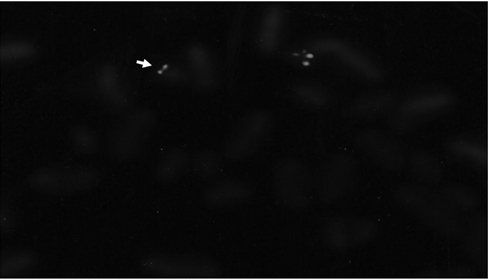
22q11.2 deletion on FISH analysis of case 2.

**Figure 3:**
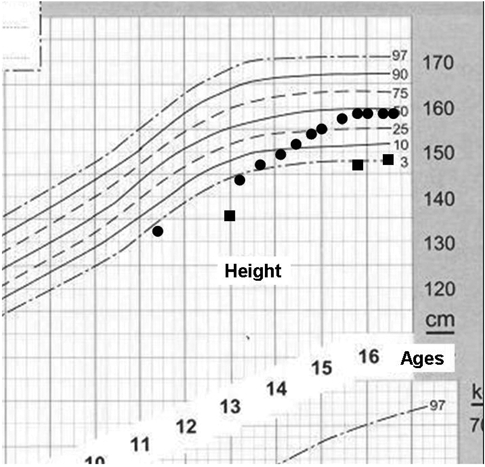
Growth chart of case 1 and 2 demonstrating growth pattern typical for constitutional delay of growth (Circles; case 1, squares; case 2).

## DISCUSSION

VCFS is a genetic syndrome with a wide range of clinical features. We report two female patients aged 11 and 13 years presenting to our pediatric endocrinology clinic, one with hypocalcaemia and short stature and, one with short stature and a severe heart defect. The facial appearance (Fig. 1a and 1b) and clinical features of these patients led us to a consider a tentative diagnosis of VCFS.

The growth pattern, age of menarche and final height of these two cases with VCFS were consistent with the pattern of growth of children with constitutional delay of growth and puberty as shown in Figure 3. Patient 1 had a satisfactory catchup growth after puberty with a final height consistent with her midparental target height. The second patient demonstrated severe short stature at presentation and also had partial GH deficiency in the first GH stimulation test.However, she demonstrated spontaneous improvement in height after puberty, suggesting that she also had a similar pattern of growth. Despite spontaneous improvement in her height SDS, this patient ended up with a short final height due possibly to intrauterine growth retardation, transient partial GH deficiency or cardiac defect.

Short stature is a frequent finding in VCFS and has been reported to occur in 36% to 67% of these patients.(13, 14, 15, 16, 17, 18, 19, 20) It was postulated to be due to intrauterine growth retardation, feeding difficulties and congenital heart defects. However, no difference in the frequency of short stature was observed in children with/or without cleft palate or congenital heart defect in VCFS (21). Patient series demonstrated that all patients with 22q.11.2 deletion and short stature were younger than 10 years of age (21), but only 10% of the adult patients were short (13). This pattern of growth is suggestive of constitutional delay growth pattern. However, in the literature available to us, we did not encounter any longitudinal growth data regarding the growth pattern of patients with VCFS. It is noteworthy that both of our patients showed growth and pubertal patterns typical for constitutional delay of growth and puberty.

Four patients with 22q11.2 deletion and short stature were described to have partial GH deficiency and abnormalities in IGFI.(22) All these cases had heights lower than −2 SDS and they all benefited from GH treatment. Our second case had severe short stature (−3.4 SDS in height) and failed in the first GH testing. However, she also showed an improvement in her height SDS after puberty and a repeat GH testing showed normal GH response to stimulation, suggesting that GH deficiency was secondary to a constitutional delay of growth and puberty. It is well known that children with constitutional delay of growth and puberty may exhibit transient GH insufficiency due to lack of priming effect of sex steroids on pituitary GH secretion.(23, 24, 25)

In our second case, short stature led to the diagnosis of VCFS with the help of additional findings which consisted of conotruncal cardiac defect, high arched palate, learning disabilities, attention deficit hyperactivity disorder, and ocular findings. Although the clinical findings are consistent with VCFS, we have not detected 22q11.2 deletion or any chromosomal anomaly in this patient. This may be due to the fact that deletion of 22q11.2 region is not detectable by extensive FISH analysis in approximately 10% of patients who are clinically diagnosed as 22q11.2 deletion and our patient may have been in this 10% group.(6, 7, 8, 9, 10, 11, 12) Another possibility in this patient could be an autosomal recessive mutation causing VCFS phenotype due to parental consanguinity.

In conclusion, the longitudinal evaluation of these two patients provide a first demonstration of a growth pattern in VCFS which is consistent with a pattern of growth typical for constitutional delay in growth and puberty. We suggest that GH stimulation tests in VCFS children should be reserved to those with height deficits exceeding –2SD of their target height.

**Fig. 1a:**
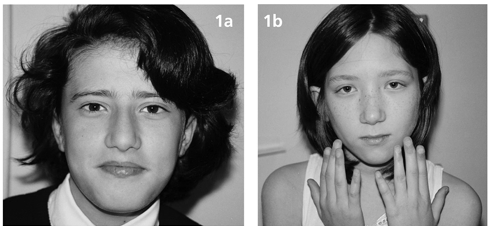
Facial appearance showing prominent nasal root, malar hypoplasia, long face, narrow palpebral fissures of case 1 (1a) and, depressed nasal bridge, hypoplastic ala nasi, long filtrum, long face, narrow palpebral fissures, hypertelorism and slender fingers of case 2 (1b).

**Figure 3:**
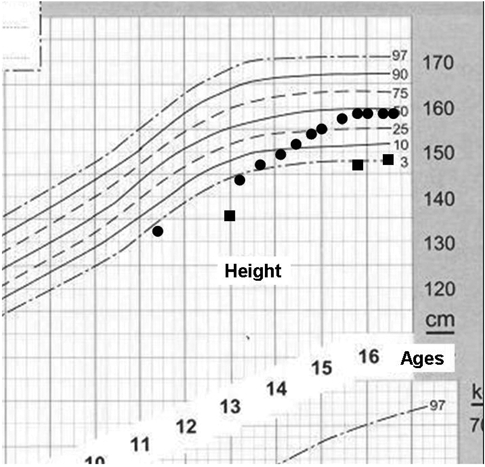
Growth chart of case 1 and 2 demonstrating growth pattern typical for constitutional delay of growth (Circles; case 1, squares; case 2).
